# Joubert syndrome with hippocampal malrotation in a 4-month-old male: A case report

**DOI:** 10.1016/j.radcr.2026.10.018

**Published:** 2026-10-30

**Authors:** Costantia A․ Rutta, Mary C․K․ Kamuzora

**Affiliations:** a Department of Radiology and Imaging, Muhimbili University of Health and Allied Sciences, Dar es Salaam, Tanzania; b Department of Radiology and Imaging, Muhimbili National Hospital - Mloganzila, Dar Es Salaam, Tanzania

**Keywords:** Joubert syndrome, Molar tooth sign, Hippocampal malrotation, Developmental delay

## Abstract

Joubert syndrome is a rare autosomal recessive ciliopathy characterized by a distinctive midbrain-hindbrain malformation, with the "molar tooth sign" as its characteristic neuroimaging hallmark, resulting from cerebellar vermian hypoplasia, thickening and horizontalization of the superior cerebellar peduncles, and a deepened interpeduncular fossa. We report a 4-month-old male infant presenting with developmental delay and progressive weight loss. Brain Magnetic Resonance Imaging revealed the characteristic molar tooth sign, vermian hypoplasia, and an associated hippocampal malrotation, a less frequently recognized but increasingly documented supratentorial finding in Joubert syndrome and related disorders. The patient also had retinal degeneration and elevated transaminases, findings that raise concern for multisystem involvement within the Joubert ciliopathy spectrum. Hippocampal malrotation has been described as an associated supratentorial finding in JS, but its clinical significance, including any relationship with epilepsy, remains uncertain. This case emphasizes careful evaluation of both infratentorial and supratentorial structures and systemic assessment in patients with JS.

## Introduction

Joubert syndrome (JS) is a rare neurodevelopmental disorder with an estimated incidence of 1 in 80,000 to 100,000 live births [1,2]. First described by Marie Joubert in 1969, the syndrome is characterized by a constellation of clinical features, including neonatal breathing dysregulation (episodic tachypnea or apnea), hypotonia, oculomotor apraxia, ataxia, and global developmental delay [2,3]. The condition is genetically heterogeneous, with over 35 identified disease-causing genes, most of which encode proteins involved in primary cilium function, placing JS within the expanding group of ciliopathies [2,4]. In some affected individuals, mutations have been mapped to chromosome 9q34.3, though many patients do not harbor abnormalities in this region.

The diagnosis of JS relies heavily on neuroimaging, with the “molar tooth sign” (MTS) on axial brain MRI considered pathognomonic [1]. This sign is produced by a complex malformation of the midbrain-hindbrain junction characterized by: (i) cerebellar vermian hypoplasia or agenesis, (ii) thickening and horizontal orientation of the superior cerebellar peduncles, and (iii) a deepened interpeduncular fossa [3]. Additional imaging features include a “bat wing” configuration of the fourth ventricle, brainstem dysmorphism, and various supratentorial anomalies [5].

While infratentorial findings are well characterized, the spectrum of supratentorial abnormalities in JSRD is increasingly recognized, including callosal abnormalities, neuronal migration disorders, and hippocampal developmental abnormalities. The literature generally describes hippocampal malrotation as incomplete hippocampal inversion with a rounded hippocampal configuration, medial positioning, and a deep or vertically oriented collateral sulcus; associated temporal horn and fornix abnormalities may be present. Its clinical significance remains uncertain [6]. The reported frequency of hippocampal malrotation varies considerably across published JSRD cohorts [4,5]. We present a case of a 4-month-old male with Joubert syndrome and bilateral hippocampal malrotation, highlighting the spectrum of supratentorial abnormalities that may accompany the characteristic midbrain-hindbrain malformation and the importance of systematic evaluation of the hippocampi on brain MRI.

## Case presentation

A case of a male infant who presented at 4 months of age with chief complaints of developmental delay and progressive weight loss. He was born via spontaneous vaginal delivery with a birth weight of 4 kg and required continous oxygen supplementation for 3 days, commencing a few hours after birth . Since birth, he has exhibited delayed milestones and failure to thrive, with poor head control, though he can coo and recognize familiar faces; however, he cannot bring his hands to his mouth and cannot fix or follow visually. His antenatal history was unremarkable, and he has four healthy siblings with no similar medical issues.

On clinical examination, he is afebrile but wasted (3.5 kg) with palmar pallor, frontal bossing, an open posterior fontanelle measuring 3 cm, a prominent occiput, hypertelorism, and repeated tongue protrusion.

His vital signs are PR 106 bpm, RR 46 breaths/min, and SpO2 98% on room air, with occasional 1–2 apneic episodes noted.

Neurological examination reveals hypotonia, reduced muscle bulk, persistently clenched fists, oculomotor apraxia with nystagmus, brisk deep tendon reflexes (lower limbs > upper limbs, grade 3), and pupils that are briskly reactive to light. No polydactyly or organomegaly were noted.

Ophthalmology assessment confirmed cortical visual impairment, retinal degeneration, nystagmus, and exposure retinopathy.

Laboratory investigations showed mild anemia, Hb 10.98 (11.1–22 g/dL), with relative lymphocytosis, monocytosis, and eosinophilia; mild neutropenia; normal WBC and platelet count. Mild hyperkalemia 5.6 (3.83–5.52 mmol/L), hypermagnesemia 0.97 (0.7–0.86 mmol/L), elevated calcium 2.87 (2.1–2.7 mmol/L); markedly elevated transaminases; AST 202.09 (0–48 U/L), ALT 138.43 (0–55 U/L); elevated vitamin D 25-hydroxy 91.7 (20–40 ng/mL); normal alkaline phosphatase, CRP, renal function, and parathyroid hormone levels.

Radiologically, abdominal ultrasound showed bilateral mildly increased renal cortical echogenicity with preserved corticomedullary differentiation; chest X-ray and hand X-ray were unremarkable. No further nephrology or hepatology evaluation was performed. Based on the clinical findings of hypotonia, developmental delay, and the history of neonatal respiratory support, an initial misdiagnosis of cerebral palsy secondary to suspected hypoxic-ischemic encephalopathy was considered. However, the presence of atypical features including hypotonia, oculomotor apraxia, nystagmus, and craniofacial dysmorphism prompted further neuroimaging evaluation. Thus, brain MRI was performed on a 3T system with sedation, utilizing standard pediatric neuroimaging sequences including sagittal T1-weighted, axial T2-weighted, axial T1-weighted, coronal T2-weighted, axial FLAIR, and diffusion-weighted imaging (DWI). The acquired voxel dimensions were 0.72 × 0.72 × 3 mm³ for the axial T2W sequences, 0.90 × 0.90 × 1 mm³ for the sagittal T1W sequence, and 0.63 × 0.63 × 3 mm³ for the coronal sequences. Additional thin section reconstructions were subsequently generated from the source data where required for detailed anatomical assessment.

### Infratentorial findings

**Midbrain:** Axial T2-weighted images at the pontomesencephalic junction demonstrated the molar tooth sign (MTS) configuration comprising thickened and elongated superior cerebellar peduncles with a more horizontal orientation and a deepened interpeduncular fossa (Fig. 1A).

**Fig. 1:**
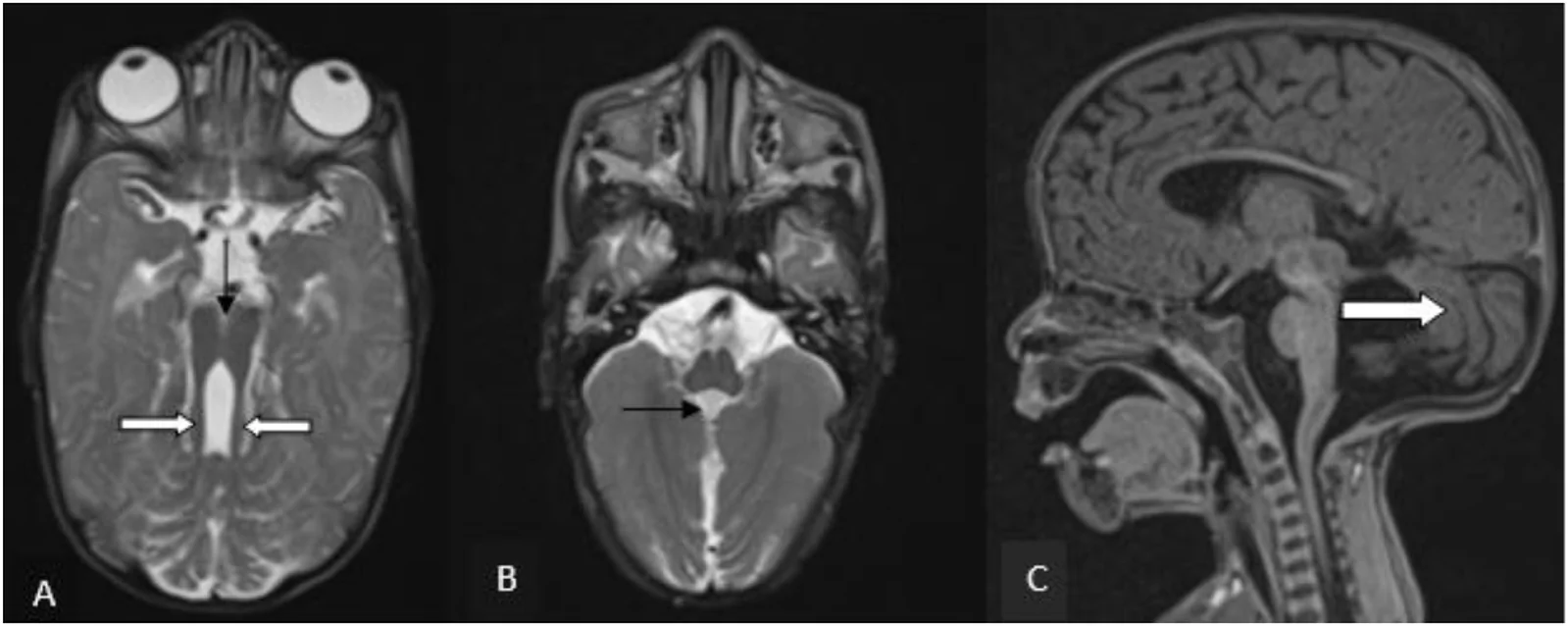
Characteristic MRI findings of Joubert syndrome. (A) Axial T2-weighted MR image at the pontomesencephalic junction demonstrates thickened, horizontally oriented superior cerebellar peduncles (white arrows) with a deepened interpeduncular fossa (thin black arrow), producing the characteristic molar-tooth appearance. (B) An axial T2-weighted MR image through the posterior fossa shows an abnormal fourth ventricle configuration associated with vermian hypoplasia (thin black arrow). (C) Sagittal T1-weighted MR image demonstrates hypoplasia of the cerebellar vermis (white arrow).

**Cerebellum and fourth ventricle:** Marked hypoplasia of the cerebellar vermis, enlargement and abnormal configuration of the fourth ventricle. The cerebellar hemispheres appeared relatively normal in size, with mild posterior fossa enlargement (Fig. 1B,C).

### Supratentorial findings

Coronal T2-weighted images demonstrated bilateral hippocampal malrotation. Both hippocampi showed a rounded rather than normal ovoid configuration, incomplete inversion, and medial positioning, with vertical orientation of the hippocampal long axis and collateral sulci. The temporal horns were also prominent(Fig. 2A-C). No abnormal hippocampal signal was identified.

**Fig. 2:**
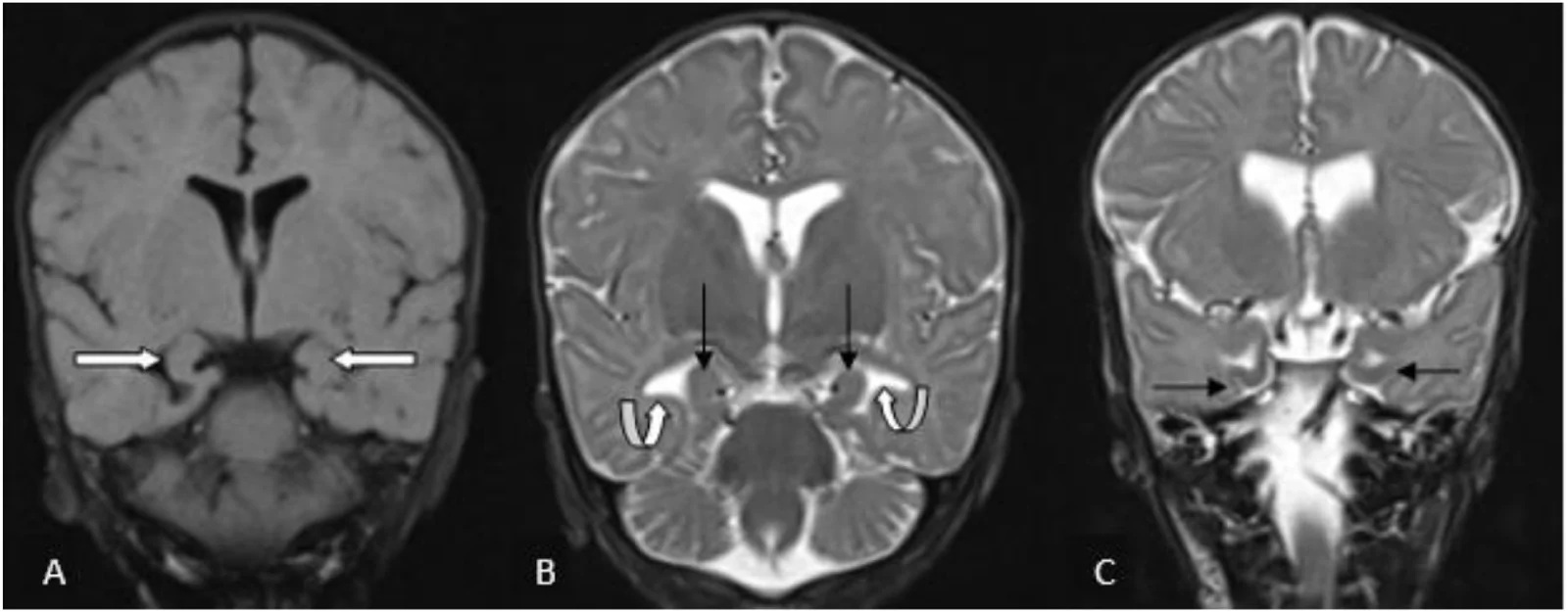
MRI features of bilateral hippocampal malrotation. (A) Coronal T1-weighted MR image demonstrates incomplete hippocampal inversion with abnormal hippocampal configuration (white arrows). (B) Coronal T2-weighted MR image demonstrates rounded, medially positioned hippocampi (curved arrows) with prominence of the temporal horns (thin black arrows). (C) Coronal T2-weighted MR image demonstrates vertically oriented collateral sulci (black arrows).

No evidence of callosal dysgenesis. No cortical migration abnormalities or polymicrogyria were identified. The myelination pattern was normal for 4 months of age.

Based on the characteristic neuroimaging findings, a diagnosis of Joubert syndrome (JS) was established. The associated hippocampal malrotation represents an additional supratentorial feature within the Joubert syndrome and related disorders (JSRD) spectrum.

The patient was discharged on medications (nutritional supplements) with a plan for visual stimulation, genetic counselling, and multidisciplinary follow-up in pediatric, ophthalmology, malnutrition, and physiotherapy clinics.

## Discussion

Joubert syndrome (JS) is a rare autosomal recessive ciliopathy, in which diagnosis relies on a combination of clinical features and characteristic neuroimaging findings, with the "molar tooth sign" (MTS) on axial brain MRI considered pathognomonic [1]. The MTS results from a complex midbrain-hindbrain malrotation characterized by cerebellar vermian hypoplasia, thickening and horizontalization of the superior cerebellar peduncles, and a deepened interpeduncular fossa [7].

Our patient presented with classic features of JS, including developmental delay, hypotonia, abnormal eye movements (nystagmus and oculomotor apraxia), and neonatal respiratory irregularities with occasional apneic episodes [8]. In addition, brisk deep tendon reflexes were consistent with the neurological phenotype described in the literature by Holla et al. [9]. The craniofacial features- frontal bossing, hypertelorism, and depressed nasal bridge align with those described by De Mori et al. (2017) [10] in SUFU-related Joubert syndrome. However, unlike their cohort, where polydactyly and polymicrogyria were common, our patient lacked these findings, underscoring the phenotypic variability in JS. The index patient also presented with ophthalmological findings of cortical visual impairment, retinal degeneration, and exposure retinopathy, some of which are documented in JSRD, with retinal dystrophy affecting up to 30% of patients [11].

The principal additional neuroimaging finding in this case was bilateral hippocampal malrotation accompanying the classic MTS. Supratentorial findings, including hippocampal malrotation, have been increasingly recognized in JSRD. Similar to our case, Poretti et al. described bilateral hippocampal malrotation in 11 of 75 patients (approximately 15%), with bilateral involvement in 3 patients [5], although some smaller studies have reported higher frequencies (Şenocak et al., 2010; 80% in a cohort of 20 patients), possibly reflecting differences in imaging protocols, sample size, or patient selection [4]. Although hippocampal malrotation has been reported in populations with seizures, its pathological and epileptogenic significance remains debated [6]. Our patient had no reported seizures; therefore, no causal or prognostic inferences regarding epilepsy can be made from this imaging finding, suggesting that hippocampal involvement may not always correlate with seizure activity.

The extracranial findings also warrant attention because Joubert syndrome is a multisystem ciliopathy. Retinal degeneration is a recognized ocular manifestation, while renal and hepatic involvement may occur within the Joubert syndrome spectrum. In this patient, retinal degeneration, increased renal cortical echogenicity, and elevated serum transaminases raise the possibility of multisystem involvement and warrant appropriate clinical correlation and longitudinal surveillance [12].

A clinical-radiological diagnosis of Joubert syndrome was made based on the characteristic molar tooth sign observed on brain MRI and the associated clinical phenotype, which included developmental delay, hypotonia, oculomotor apraxia, and neonatal respiratory irregularities. Molecular genetic confirmation was not performed due to limited access to genetic testing facilities at our institution. Nevertheless, the molar tooth sign, in association with the characteristic clinical features, is considered sufficient to establish a clinical diagnosis of Joubert syndrome even in the absence of molecular genetic confirmation [12].

Genetic counselling is essential for the family, given the autosomal recessive inheritance pattern and the 25% recurrence risk for future pregnancies [4]. The identification of the causative mutation, if pursued, would allow for early prenatal diagnosis in at-risk couples. The multidisciplinary management approach involving pediatrics, ophthalmology, malnutrition management, and physiotherapy is crucial for optimizing outcomes in this complex disorder [13].

A focused literature search did not identify a previously published East African case specifically describing Joubert syndrome with hippocampal malrotation. Therefore, to the authors’ knowledge, this may represent the first such case reported from East Africa, underscoring the value of this report in expanding regional awareness of the neuroimaging spectrum of JSRD and highlighting the diagnostic feasibility of this condition even in resource-limited settings where genetic confirmation is not readily accessible.

## Learning points

This case offers several important learning points for clinicians and radiologists managing infants with neurodevelopmental delays.

First, Joubert syndrome should be considered in the differential diagnosis of any infant presenting with neonatal respiratory irregularities, hypotonia, developmental delay, and abnormal eye movements, as early recognition allows for timely multidisciplinary intervention.

Second, the molar tooth sign on axial brain MRI is the pathognomonic imaging hallmark of JS, and its identification should prompt a systematic search for associated supratentorial anomalies, including hippocampal malrotation, whose clinical significance remains uncertain.

Third, because JS is a multisystem ciliopathy, patients require comprehensive evaluation for renal, hepatic, ocular, and skeletal involvement, which should prompt targeted assessment and longitudinal follow-up.

Fourth, this case highlights the critical importance of neuroimaging in preventing diagnostic errors, as the patient was initially misdiagnosed with cerebral palsy based on clinical findings alone, emphasizing that cerebral palsy is a clinical diagnosis that requires careful evaluation, particularly when atypical presentations or associated systemic features should prompt further investigation with brain MRI.

Finally, the autosomal recessive inheritance pattern necessitates genetic counselling for the family, with a 25% recurrence risk and the potential for prenatal diagnosis in subsequent pregnancies. Early and accurate diagnosis through comprehensive neuroimaging is therefore essential for guiding appropriate management and genetic counselling in patients with Joubert syndrome and related disorders.

## Patient consent

Written consent was obtained from the patient's parents for publication of this case report and accompanying images.
